# Production Systems and Age Influence Fecal Mycobiota Diversity and Composition in Swine

**DOI:** 10.1007/s00248-025-02614-0

**Published:** 2025-10-02

**Authors:** Carolyn M. Scott, Devin B. Holman, Katherine E. Gzyl, Angela Ibe, Ahmad Esmaeili Taheri

**Affiliations:** 1 Red Deer Polytechnic, Red Deer, AB Canada; 2 Lacombe Research and Development Centre, Agriculture and Agri-Food Canada, Lacombe, AB Canada

**Keywords:** Pigs, Fungi, Mycobiota, ITS sequencing, Pasture, Microbiota

## Abstract

The gut microbiome is an important factor in animal health and can be influenced by factors such as age, diet, stress, environmental conditions, and farming practices. Bacterial communities of the gut microbiome in many species have been extensively studied, but research on the fungal microbiota remains limited and underrepresented in the literature. The objective of this study was to characterize the fecal mycobiota of swine raised under two different production systems: outdoor pasture-based or conventional indoor systems. Fecal samples from nursery, growing-finishing, and sow pigs from both farming systems were collected, and the mycobiota was profiled using PCR amplification and sequencing of the universal fungal internal transcribed spacer 1 (ITS1) region. A significant difference in fungal community structure was observed between the conventionally raised and pasture-raised pigs, as well as among all three production phases. Four species, *Arthrographis kalrae*, *Enterocarpus grenotii*, *Pseudallescheria angusta*, and *Sagenomella oligospora*, were differentially abundant between the two farms, all of which had higher relative abundance in the pasture-raised pigs. Additionally, pasture-raised pigs hosted a more diverse fungal community with higher species richness in their gastrointestinal tract. In summary, farming practices and pig age influenced the pig fecal mycobiota.

**Supplementary Information:**

The online version contains supplementary material available at 10.1007/s00248-025-02614-0.

## Introduction

The gut microbiome of mammals is highly diverse, comprising various types of microorganisms, including bacteria, archaea, viruses, protists, and fungi [1–3]. These microorganisms play key roles in the host’s physiology and overall health [1, 3, 4] and are influenced by factors such as age, diet, and lifestyle [1, 3]. Studies have shown that the mycobiota in the gut of mammals may contribute to maintaining homeostasis [5, 6], aid in the digestion of food, especially proteins [7], regulate immune cell responses [8, 9], and associate with certain diseases [10]. In pigs specifically, the gut mycobiota is thought to consist of several commensal species [3, 11, 12]. For example, one study demonstrated that *Kazachstania slooffiae* promotes the production of biofilms in the gastrointestinal (GI) tract [11]. Another study demonstrated that dietary supplementation with *Saccharomyces cerevisiae* can improve the performance of piglets [12]. However, mycotoxins produced by certain fungal species can contaminate pig feed, adversely affecting piglet development and sow reproduction [13].

Farming practices can significantly influence the composition of fungal communities in the GI tract. Conventionally raised pigs are typically housed indoors [14] in large numbers on concrete or rubber floors. Conversely, pasture-raised pigs are reared outdoors in smaller densities [14], with access to soil and native vegetation, allowing them to graze [14, 15]. Conventional systems also have more rigid practices for managing pigs of different ages. For example, piglets are weaned and separated from their mothers at a relatively early age [14] and then housed in pens with other individuals of the same age. Pigs raised under these two different systems have previously been shown to have significantly different fecal bacterial communities [16].

Research on the pig gut microbiota has primarily focused on bacterial communities, while our understanding of fungal communities remains limited. Understanding the factors that shape the mycobiota is important, as fungi may influence gut health, immune responses, and ultimately animal productivity. In pigs, two major factors that may influence fungal communities are age and management system, both of which are central to commercial production. Therefore, the objective of this study was to characterize the fungal communities in the feces of pigs raised under conventional indoor and pasture-based systems across three production stages. Identifying management factors associated with the mycobiota can provide insights that may guide husbandry practices aimed at improving swine health and performance. It was hypothesized that, similar to bacterial communities, the fecal mycobiota would differ significantly between conventionally and pasture-raised pigs, and that distinct fungal communities would be found at different production stages.

## Methods

### Animals and Sample Collection

Fecal samples were collected from the same pigs described in an earlier study [16]. Briefly, conventionally and pasture-raised pigs from three production phases were sampled using sterile gloves and fecal swabs (FLOQSwabs; Copan, Murrieta, CA, USA). Conventional: nursery (*n* = 6), growing-finishing (*n* = 10), and sows (*n* = 12); pasture: nursery (*n* = 10), growing-finishing (*n* = 6), and sows (*n* = 13). All fecal samples were immediately placed on ice, transported to the laboratory, and stored at − 80 °C until DNA extraction.

The conventionally raised sows were Landrace × Yorkshire, and the nursery and growing-finishing pigs were Landrace × Yorkshire × Duroc. These pigs were housed in pens within a farrow-to-finish barn on concrete slatted floors, and piglets were weaned at 21 days of age. The pasture-raised pigs were crossbred Duroc, Tamworth, Berkshire, and Large Black. They were housed outdoors year-round without confinement, did not receive antimicrobials, and had continuous access to pasture throughout the production cycle. Piglets on the pasture farm were weaned at 8 weeks of age and subsequently grouped with pigs of similar age. Pigs on both farms received a grain-based diet consisting primarily of wheat and barley. However, unlike the conventional pigs, the diet for the pasture-raised pigs did not include canola, corn, or soy. The two farms were located within 200 km of each other in Alberta, Canada.

### DNA Extraction, ITS1 Sequencing, and Data Analysis

DNA was extracted from the fecal samples as outlined in Holman et al. [16], and the internal transcribed spacer 1 (ITS1) region was amplified using PCR. The following primers were used: ITS1-F, 5′-CTTGGTCATTTAGAGGAAGTAA-3′ [17], and ITS2-R,

5′-GCTGCGTTCTTCATCGATGC-3′ [18]. The PCR master mix consisted of 2.5 µL of 10X buffer with 15 mM MgCl_2_ (Qiagen Inc., Hilden, Germany), 1.25 µL dimethyl sulfoxide, 0.5 µL of 10 mM dNTPs (New England Biolabs, Ipswich, MA, USA), 0.1 µL HotStarTaq (5U/µL) (Qiagen Inc.), 0.6 µM of each primer, 10 ng of DNA template, and 19.35 µL molecular grade water in a total volume of 25 µL. The thermocycler conditions were: an initial denaturation at 96 ℃ for 15 min, followed by 33 cycles of 96 ℃ for 45 s, 52 ℃ for 45 s, and 72 ℃ for 60 s, with a final extension at 72 ℃ for 10 min. Indices were then added to the amplicons via a second round of PCR. The amplicons were quantified using a Quant-iT PicoGreen dsDNA Assay Kit (Thermo Fisher Scientific, Mississauga, ON, Canada), pooled, and then purified using AMPure XP beads (Beckman Coulter, Mississauga, ON, Canada). The purified ITS1 amplicon libraries were sequenced on a MiSeq instrument (600 cycles) (Illumina Inc., San Diego, CA, USA) with a v3 reagent kit (Illumina Inc.).

Primer sequences were removed from the reads using Cutadapt v.2.10 [19]. DADA2 v.1.32.0 in R v.4.4.0 was then used to filter out low-quality sequences using default parameters and a minimum read length of 50 bp. The resulting reads were then denoised and merged, and screened for chimeras, which were subsequently removed. Taxonomy was assigned to the resulting amplicon sequence variants (ASVs) using the naïve Bayesian classifier and the UNITE (v.10.0) [20] database. Prior to the calculation of alpha and beta diversity metrics, all samples were rarefied to 7500 sequences to account for differences in sequencing depth. Four DNA extraction control samples were also sequenced, with one ASV classified as *Trametes versicolor* removed from the biological samples based on its high abundance in the extraction controls.

### Statistical Analysis

The number of observed ASVs (richness) and the inverse Simpson diversity index values were compared between conventionally raised and pasture-raised pigs, with production phase included in the model, using a type III two-way analysis of variance (ANOVA), which is appropriate for unbalanced designs. A Box–Cox transformation was applied to these measures to improve normality, and the normality of residuals was subsequently confirmed with the Shapiro–Wilk test. Differences in fungal community structure were evaluated using Bray–Curtis dissimilarities and permutational multivariate analysis of variance (PERMANOVA) with vegan v.2.6.10 and phyloseq v.1.50.0 in R, with both farm type and production phase included in the model. Pairwise comparisons between farm types were further conducted within each production phase using the pairwiseAdonis package v.0.4.1 (https://github.com/pmartinezarbizu/pairwiseAdonis). Differentially abundant species in the fecal mycobiota of conventionally raised and pasture-raised pigs were identified with MaAsLin2 (v.1.20.0) [21].

## Results

### Fecal Mycobiota Summary

The sequencing yielded 31,305 ± 1507 (mean ± SEM) ITS1 reads per sample. These sequences were assigned to 2135 amplicon sequence variants (ASVs) representing 6 phyla, 216 genera, and 312 species. In both the conventionally raised and pasture-raised pigs, nearly all sequences (99.4%) were assigned to ASVs classified as members of the Ascomycota or Basidiomycota phyla (data not shown). In total, 76.9% ± 2.3% and 93.6% ± 1.4% of the ITS1 reads were assigned to a specific species or genus, respectively.

### Fecal Mycobiota of Conventionally Raised vs. Pasture-Raised Pigs

**Fig. 1:**
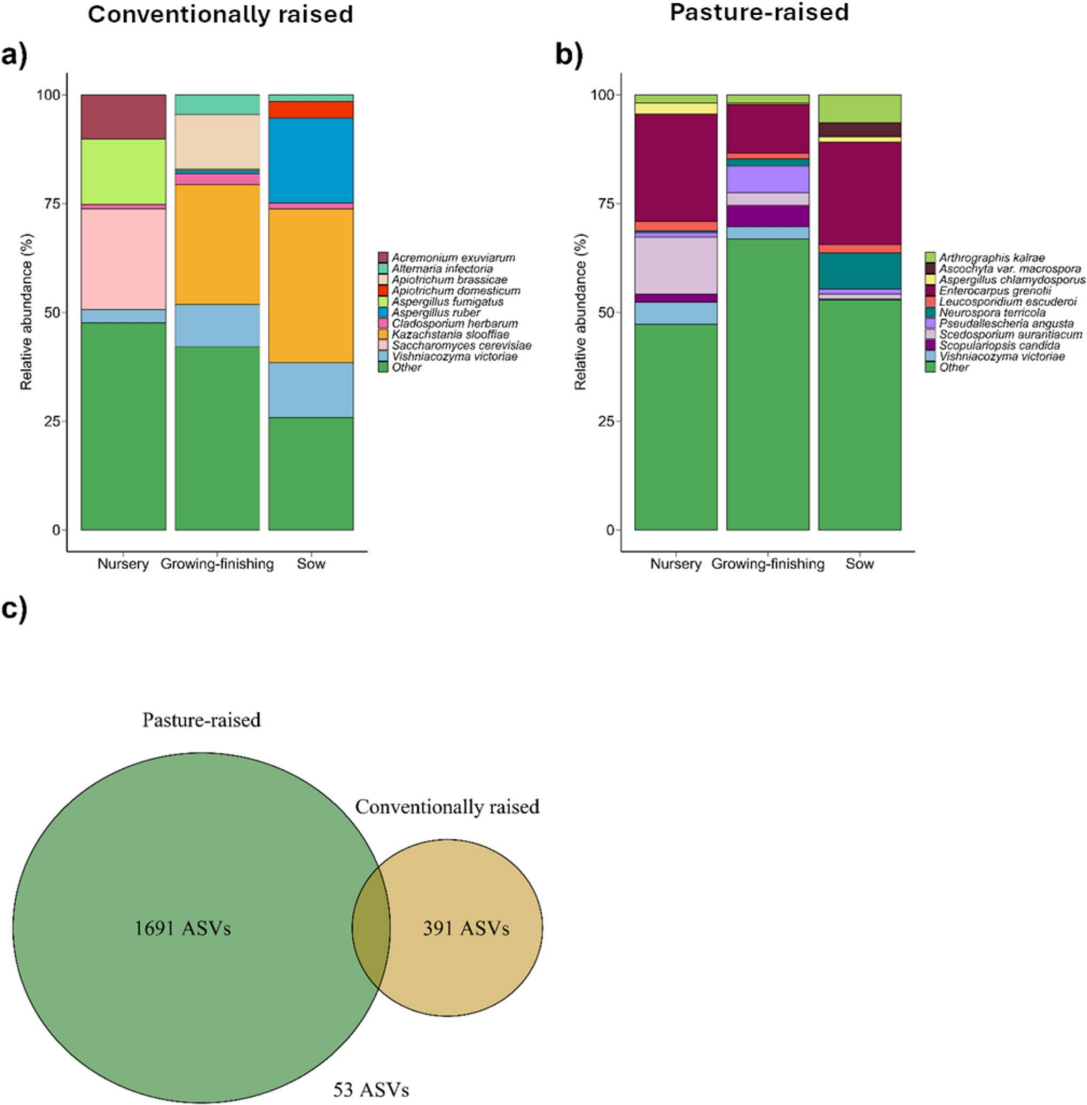
Stacked bar plots showing the ten most relatively abundant species in the gut mycobiota of (**a**) conventionally raised (*n* = 28) and (**b**) pasture-raised (*n* = 29) pigs across three production phases: nursery, growing-finishing, and sows. (**c**) Venn diagram depicting the number of amplicon sequence variants (ASVs) unique to the pasture-raised and conventionally raised pigs, as well as those shared between the two groups

The ten most relatively abundant species in the conventionally raised and pasture-raised pig fecal mycobiota showed little overlap with each other, with the exception of *Vishniacozyma victoriae*, which was relatively abundant in both farms (mean across farms: 5.9% ± 1.4%) (Fig. 1). In addition to *V*. *victoriae*, *Enterocarpus grenotii* and *Scedosporium aurantiacum* were among the species with the highest relative abundances in the pasture pig fecal mycobiota, while *K. slooffiae* and *Aspergillus ruber* were relatively abundant in the conventional pigs*.* In most cases, the relative abundance of these species varied by production phase within each farm (Supplementary Table S1). For example, although relatively abundant overall, *Saccharomyces cerevisiae* was only identified in the conventionally raised nursery pigs, with the exception of one conventional sow sample. Only 57 out of the 2135 total ASVs were detected in at least one fecal sample from both farms (Fig. 1c), and none was identified in more than 50% of all samples (data not shown).

**Fig. 2:**
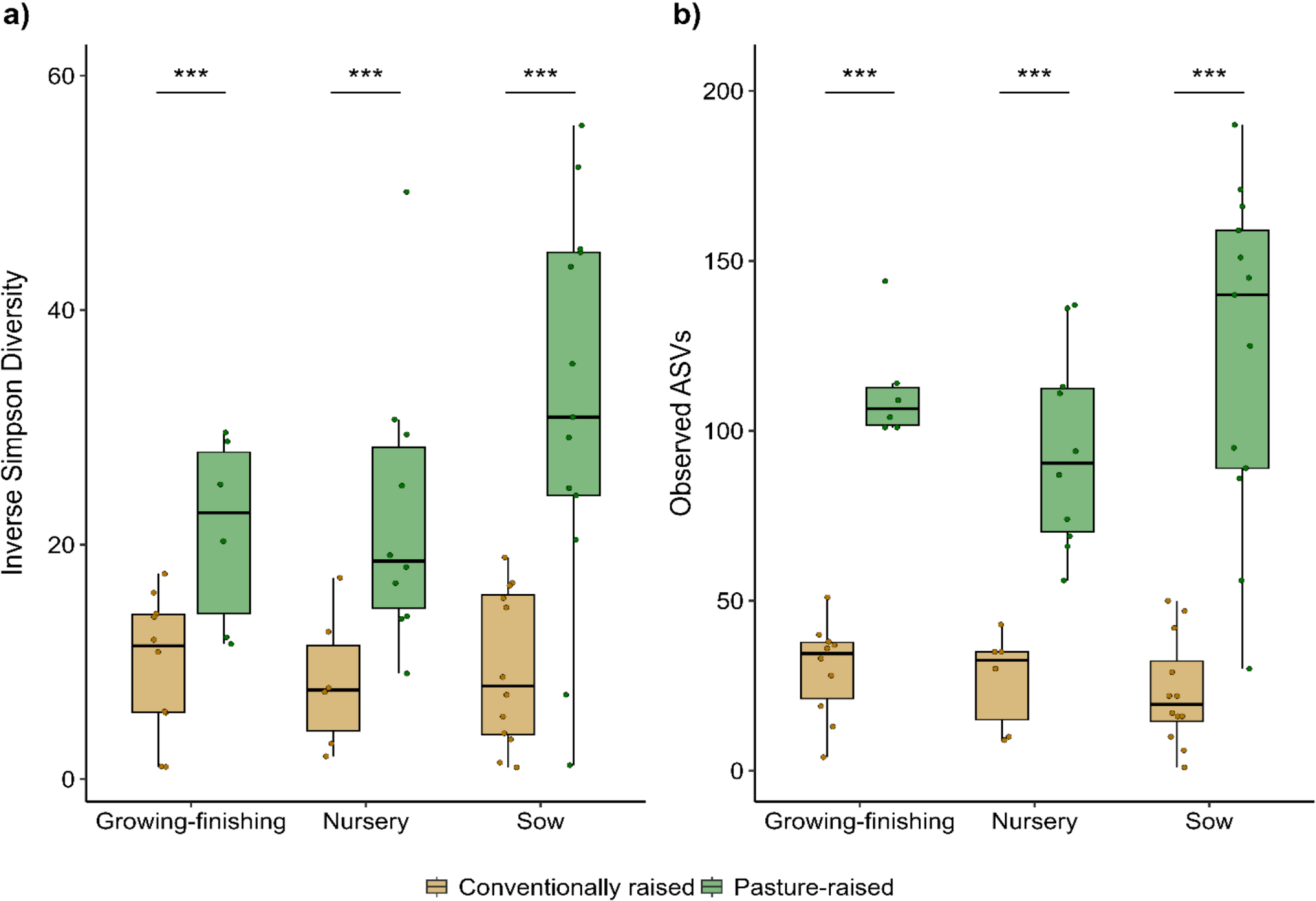
Alpha diversity measures for the fecal mycobiota of conventionally (*n* = 28) vs. pasture-raised (*n* = 29) pigs at three production phases. (**a**) Number of observed amplicon sequence variants (ASVs) and (**b**) inverse Simpson diversity index. *** indicates *P* < 0.001. The interquartile range (IQR) (middle 50% of the data), the median value, and the whiskers representing 1.5 times the IQR are displayed

**Fig. 3:**
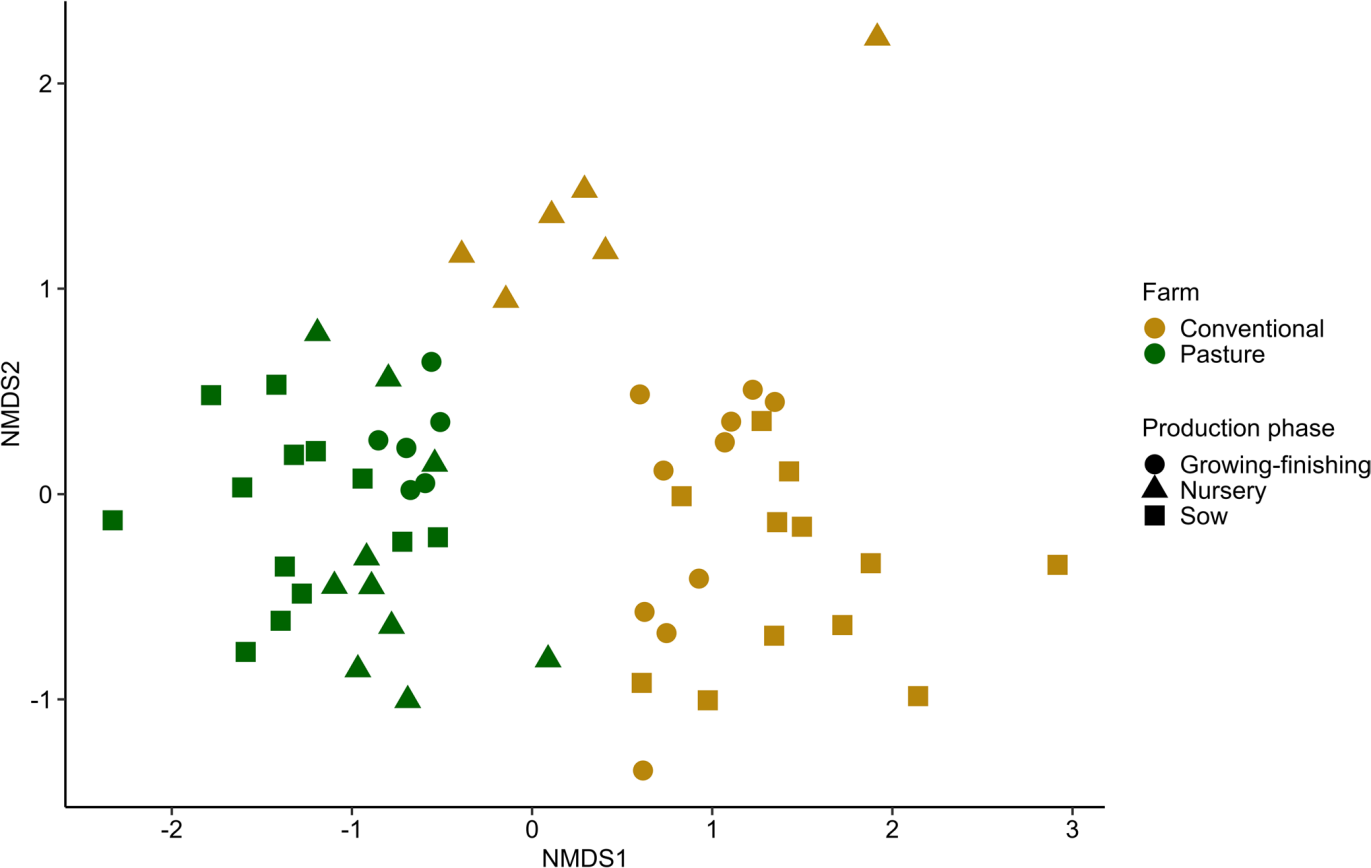
Non-metric multidimensional scaling (NMDS) plot of Bray–Curtis dissimilarities of the gut mycobiota of conventionally raised (*n* = 28) vs. pasture-raised (*n* = 29) pigs across three production phases: nursery, growing-finishing, and sows

The pasture-raised pigs had greater fungal diversity and richness in their fecal mycobiota compared with conventionally raised pigs (Fig. 2; *P* = 4.7 × 10^−12^ to 7.2 × 10^−6^). The pigs on the two farms also had significantly different fecal mycobiota based on Bray–Curtis dissimilarities (Fig. 3; PERMANOVA: *R*^2^ = 0.07, *P* < 0.001). Production phase also explained a significant portion of the variation (*R*^2^ = 0.06, *P* < 0.001). Within each production phase, differences between farms were most pronounced in the growing–finishing pigs (*R*^2^ = 0.17, *P* = 0.003), followed by the nursery pigs (*R*^2^ = 0.14, *P* = 0.02), and the sows (*R*^2^ = 0.13, *P* = 0.001) (Supplementary Table S2).

**Fig. 4:**
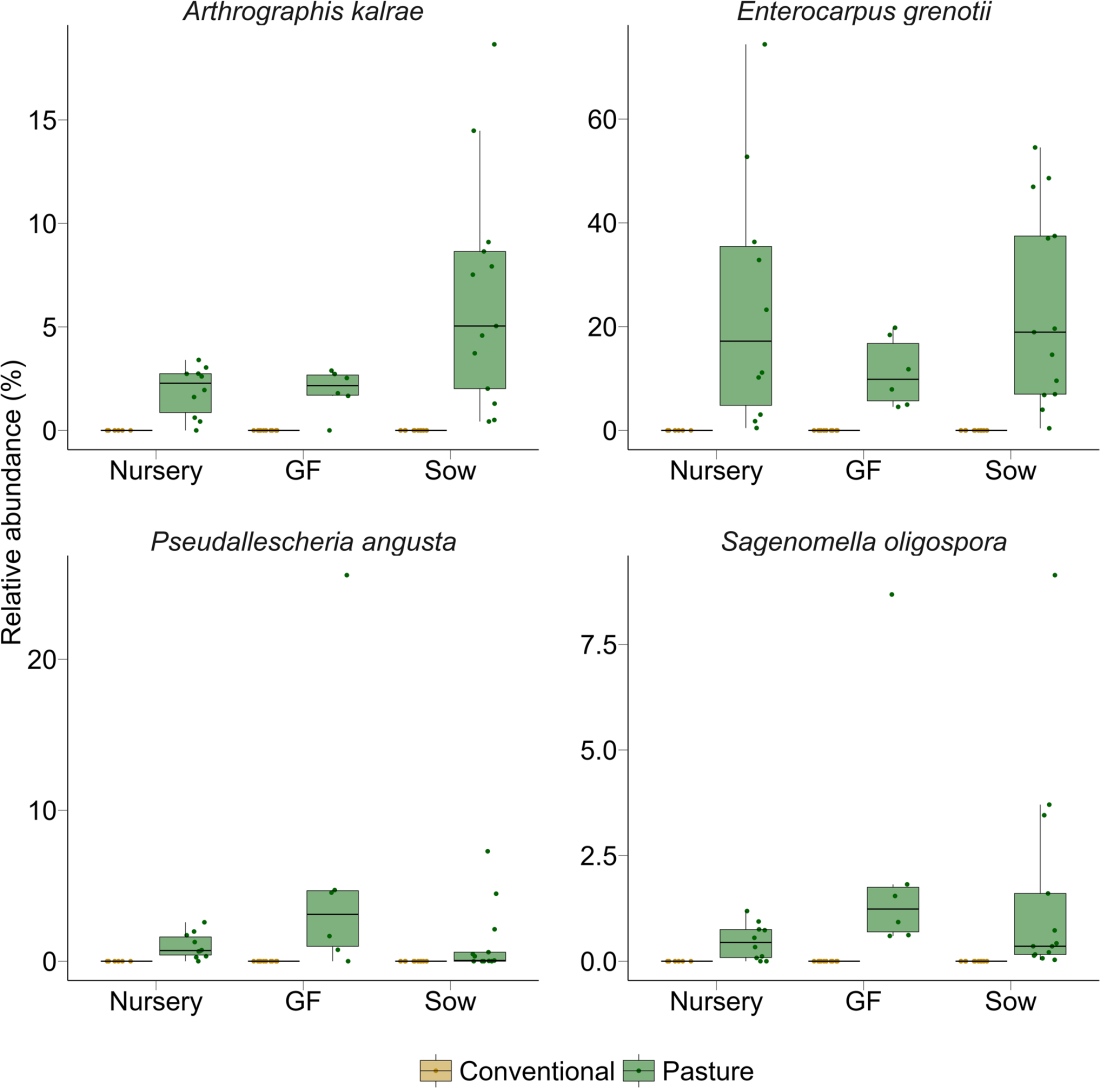
Box plots of the relative abundance (%) of the four consistently differentially abundant species between the conventionally raised (*n* = 28) and pasture-raised (*n* = 29) pigs by production phase. The interquartile range (IQR) (middle 50% of the data), the median value, and the whiskers representing 1.5 times the IQR are displayed. GF: growing-finishing

Differentially abundant species were identified between the two farms within each production phase using MaAsLin2 (Supplementary Table S3). In the nursery phase, 10 fungal species were differentially abundant, including *Aspergillus fumigatus* and *S*. *cerevisiae*, which were more abundant in conventionally raised pigs, and *E*. *grenotii* and *Aspergillus chlamydosporus*, which were enriched in pasture-raised pigs. In the growing-finishing phase, *K. slooffiae* and *Apiotrichum brassicae* were more abundant in conventional pigs, whereas 15 species, including *Mycothermus thermophilus* and *Tausonia pullulans*, were enriched in pasture-raised pigs*.* Among the 30 differentially abundant species in sows were *K*. *slooffiae* and *V. victoriae* in conventionally raised sows and *L. escuderoi* and *E. grenotii* in sows raised on pasture (Supplementary Table S3). Four fungal species differed significantly between farms across all three production phases: *Arthrographis kalrae*, *E. grenotii*, *Pseudallescheria angusta*, and *Sagenomella oligospora* (Fig. 4)*.* Notably, all four were detected exclusively in pasture-raised pigs and were absent from the fecal mycobiota of the conventionally raised pigs.

## Discussion

Given the commercial value of the swine industry, it is important to understand how the gut mycobiota is impacted by different management practices. As such, in the present study, we characterized the fecal mycobiota of conventionally raised and pasture-raised pigs across three common production phases. Significant differences were observed between the two groups, as well as across the nursery, growing-finishing, and sow production phases. In particular, the pasture pigs had greater fungal diversity and richness compared to the conventional pigs. The most relatively abundant fungal species in pigs from each farm also differed substantially. Additionally, four species, *A*. *kalrae*, *E*. *grenotii*, *P*. *angusta*, and *S*. *oligospora*, were determined to be significantly differentially abundant across the three production phases, and all were exclusive to the pasture-raised pigs. Each of these species has previously been isolated and identified in soil samples [22–29]. Pigs are known to root in soil with their snouts [14], ingesting soil and plant matter in the process [15]. Given that all these species were found exclusively in pasture-raised pigs and were not detected in the conventionally raised pigs, it is likely that the pigs on pasture acquired these fungi from the external environment.

The gut mycobiota of all pigs was dominated by the Ascomycota and Basidiomycota phyla, which is consistent with previous studies on the pig gut mycobiota [30, 31]. The yeast *V. victoriae*, a member of the Basidiomycota, was among the relatively abundant species in the fecal mycobiota of both conventionally raised and pasture-raised pigs. This species has previously been identified in pigs in Western Canada [32] and in wheat kernels grown in the Canadian prairies [33]. Given that pigs on both farms were fed a grain-based diet, this may explain the consistent presence of *V. victoriae* in both groups. *Scedosporium aurantiacum*, detected in all three production phases of pasture-raised pigs, is a known opportunistic pathogen in humans [34, 35]. Previous research has associated *S. aurantiacum* with several human diseases and infections, including scedosporiosis and cystic fibrosis [34, 35]. This species has been widely found in soil samples in numerous countries, including Canada [25, 35], and has been isolated from soil collected from drainage areas on a pig farm in Thailand [34]. Similarly, *Scopulariopsis candida*, which was relatively abundant in the pasture-raised pigs, is also predominantly found in soil and has been linked to invasive sinonasal disease in humans [36]. *Neurospora terricola,* another species that was relatively abundant in the pasture pig fecal mycobiota, has been discovered in healthy soil [29] and windblown dust samples from farms using biosolids rather than synthetic fertilizers [37].

*Alternaria infectoria,* which was among the ten most relatively abundant species in conventional pigs, is also a known human pathogen. For example, this species has been implicated in cases of cerebral phaeohyphomycosis [38, 39]. Interestingly, a study in mice found that, despite being phagocytized by macrophages, the conidia of *A. infectoria* were still able to germinate within the macrophages [39]. *Alternaria* spp. are known to produce mycotoxins, and *A. infectoria* strains isolated from winter wheat have been found to produce seven mycotoxins [40]. *Aspergillus* spp. are well known for their roles as both allergens and pathogens [41–43]. Two *Aspergillus* spp., *A. ruber* and *A. fumigatus,* were among the relatively abundant species in the conventionally raised sows and nursery pigs, respectively. Another member of the *Aspergillus* genus, *A. chlamydosporus*, was relatively abundant in the fecal mycobiota of pasture-raised pigs. Previous studies have identified *A. fumigatus* in straw and settled dust within a finishing pig stable [41], as well as in healthy soils [29]. This species is generally recognized as an allergen and opportunistic pathogen, capable of causing invasive infections in humans [42, 43]. Another species among the most relatively abundant fungi in the feces of conventional pigs was *Cladosporium herbarum*, which is also a well-known allergen [43] that has been isolated from aerosols in swine barns [44].

In conventionally raised pigs, *K. slooffiae* was relatively abundant within the sows and the growing-finishing pigs. Interestingly, with the exception of one pig, this species was not detected in the feces of conventional nursery pigs (7 days post-weaning) or any of the pasture-raised pigs, suggesting that *K. slooffiae* is well-adapted to the GI tract of pigs raised indoors on solid feed for a period of time. In agreement with this, *K. slooffiae* has been reported by others to be predominant in the GI tract of post-weaned commercial pigs [31, 32] and is suspected to have a commensal relationship with the host as well as with bacteria in the gut microbiome. For example, Summers et al. [11] discovered that *K. slooffiae* biofilm formation was enhanced in the presence of *Lactobacillus acidophilus* isolated from the swine GI tract. Additionally, *K*. *slooffiae* has been shown to decrease lysine desuccinylation and increase ATP production via glycolysis in porcine intestinal cells in vitro [31]. For these reasons, *K*. *slooffiae* has been assessed as a potential probiotic in pigs, though no measurable effects on growth performance or health have been observed to date [45, 46].

In contrast to *K. slooffiae*, the yeast species *S. cerevisiae* was relatively abundant in the fecal mycobiota of conventionally raised nursery pigs. Similar to *K. slooffiae*, *S. cerevisiae* has been investigated as a potential probiotic in pigs, with increased average daily gain observed in pre-weaned piglets receiving *S*. *cerevisiae* via oral gavage [12]. Additionally, both pre- and post-weaning dietary supplementation with *S*. *cerevisiae* have been shown to reduce the duration and severity of diarrhea in pigs challenged with enterotoxigenic *Escherichia coli* [47]. Furthermore, a *S. cerevisiae* strain isolated from the pig GI tract inhibited pathogenic *Enterobacteriaceae* species through co-aggregation [48]. In mice, dietary supplementation with *S. cerevisiae* has also been shown to stimulate phagocytic activity and other immune cells in the intestine [8, 49]. A previous study comparing fecal mycobiota across various age groups of commercial and feral pigs reported findings similar to those of the present study, with feral pigs having a richer and more complex gut mycobiota than commercial pigs [32]. Although the feral pigs in the aforementioned study are quite different from the pasture-raised pigs here, they share some environmental similarities, particularly their exposure to fungal species associated with native vegetation and soil. As such, compared to the feral pigs, the commercial pigs in the work by Prisnee et al. [32] had a higher abundance of species in the *Kazachstania* and *Saccharomyces* genera, both of which were also among the relatively abundant taxa in the conventionally raised pigs in the current study.

Future research should focus on the specific impacts of individual fungal species on the health and development of pigs. Although farm type clearly influenced the fecal fungal community composition, it explained only a modest proportion of the overall variation (*R*^2^ = 0.07), suggesting that additional drivers such as age-related physiological changes, inter-individual variation among animals, and microenvironmental exposures within farms likely also shape the fecal mycobiota. Some of the species detected in the present study may be pathogenic, while others may confer beneficial effects on the host; however, their full impacts remain unknown or understudied. Understanding the positive and negative effects of specific fungal species on host physiology could provide valuable insights for improving the health and productivity of commercial pigs.

## Conclusion

In summary, the gut mycobiota of pigs is strongly influenced by management strategies and environmental conditions under which they are raised. Pasture-raised and conventionally raised pigs exhibited significantly different gut fungal community compositions. Moreover, pasture-raised pigs had a significantly more diverse and richer gut mycobiota compared to conventionally raised pigs, likely due to their exposure to a broader range of fungal species in their environment.

## Supplementary Information

Below is the link to the electronic supplementary material.

ESM 1 (35.5 KB XLSX)

## Data Availability

The ITS1 reads are available in the NCBI sequence read archive under accessions: SRR33474760-SRR33474788 and SRR33476398-SRR33476425.
